# Software for subjective visual vertical assessment: an observational cross-sectional study

**DOI:** 10.5935/1808-8694.20120008

**Published:** 2015-11-20

**Authors:** Theo Zeferino Pavan, Martha Funabashi, José Ailton Oliveira Carneiro, Taiza Elaine Grespan dos Santos Pontelli, Walfred Tedeschi, José Fernando Colafêmina, Antonio Adilton Olivera Carneiro

**Affiliations:** a MSc. (PhD student in the Department of Physics, School of Philosophy, Sciences, and Literature of Ribeirão Preto, University of Sao Paulo); b MSc. (PhD student in the Rehabilitation Sciences Department, School of Rehabilitation Medicine, University of Alberta); c MSc. (PhD student in the Department of Medical Practice, Medical School of Ribeirão Preto, University of Sao Paulo); d PhD (PhD student in the Department of Neurosciences and Behavior Sciences, Medical School of Ribeirão Preto, University of Sao Paulo); e PhD (Hospital das Clínicas, Medical School of Ribeirão Preto, University of Sao Paulo); f PhD, Assisting Professor (Professor in the Department of Ophthalmology, Otorhinolaryngology, and Head and Neck Surgery, Medical School of Ribeirão Preto, University of Sao Paulo); g PhD (Professor in the Department of Physics, School of Philosophy, Sciences, and Literature of Ribeirão Preto, University of Sao Paulo)

**Keywords:** saccule and utricle, software design, vestibular function tests, vestibule, labyrinth

## Abstract

Spatial orientation in relation to the gravitational axis is significantly important for the maintenance of the posture, gait and for most of the human's motor activities. The subjective visual vertical exam evaluates the individual's perception of vertical orientation.

**Objectives:**

The aims of this study were (1) to develop a virtual system to evaluate the subjective visual vertical exam, (2) to provide a simple tool to clinical practice and (3) to assess the subjective visual vertical values of h ealthy subjects using the new software. Study Design: observational cross-sectional study.

**Methods:**

Thirty healthy volunteers performed the subjective visual vertical exam in both static and dynamic conditions. The exam consisted in adjusting a virtual line in the vertical position using the computer mouse. For the static condition, the virtual line was projected in a white background. For the dynamic condition, black circles rotated in clockwise or counterclockwise directions. Six measurements were taken and the mean deviations in relation to the real vertical calculated.

**Results:**

The mean values of subjective visual vertical measurements were: static −0.372°; ± 1.21; dynamic clockwise 1.53° ± 1.80 and dynamic counterclockwise −1.11° ± 2.46.

**Conclusion:**

This software showed to be practical and accurate to be used in clinical routines.

## INTRODUCTION

The spatial orientation in relation to the Earth's gravitational axis is significantly important for the maintenance of the posture, gait and for most of the human's motor activities. This spatial orientation is done through the integration of four different sensory inputs: the interoceptive, visual, somatosensory and vestibular systems^1^, ^2^, ^3^, ^4^, ^5^, ^6^, ^7^, ^8^.

Generally, this multisensory integration presents several recognized benefits such as the improvement in accuracy, precision or reaction times promoted by the simultaneous presentation of two or more sensory cues during sensory discrimination tasks. In addition, the information provided by one single sensor is often ambiguous and can be resolved only by combining cues from multiple sensory sources^5^^,^^9^. Specifically, the involvement of this multisensory integration in the representation of verticality has been suggested and the role of visual and vestibular information in verticality perception has been widely investigated^5^^,^^7^^,^^8^^,^^10^, ^11^, ^12^, ^13^.

The perceptions that represent the subjective spatial perceptions of verticality are evaluated by the subjective haptic vertical, subjective postural vertical, subjective straight ahead and subjective visual vertical (SVV)^14^, ^15^, ^16^, ^17^, ^18^. The subjective haptic vertical is determined by manipulating a wooden or metal bar into the earth-vertical position with the subjects' eyes closed. The subjective haptic vertical is driven by haptic perception originated from the stimulation of mechanoreceptors in the skin, muscles, tendons and joints in the process of the manual exploration of the bar in space^7^^,^^14^^,^^15^. The subjective postural vertical is assessed with the subjects seated on a tiltable chair that is capable of rotating in a particular plane and is immobilized by lateral stabilization to prevent postural reactions. The subjects inform, in absence of vision, when they feel their body oriented in the vertical position. The subjective postural vertical relies on information originated from graviceptors of the trunk and also from information from head and neck receptors^16^, ^17^, ^18^. The subjective straight ahead is assessed by asking the subjects to point to the position they perceive as straight ahead and represents an egocentric reference framework^19^^,^^20^. Finally, the SVV is assessed by asking the subject to align a luminous bar in the vertical position, without any reference of the real vertical, in the complete darkness^1^^,^^21^^,^^22^.

The SVV is a valid clinical exam and the deviations of the luminous bar in relation to the gravity's vertical axis are measured in degrees^2^, ^3^, ^4^, ^5^, ^6^. This capacity to judge whether the bar is aligned with the real vertical or not, depends on the integrity of visual and vestibular otolithic information^3^, ^4^, ^5^, ^6^^,^^11^^,^^12^^,^^15^^,^^22^^,^^23^. In the visual information, there is a dissociation of ventral and dorsal processing streams based on the neural mechanisms involved in judging the identity or location of a target, respectively^18^^,^^24^. In the visual cortex, the orientation preference of cells are systematically organized. The cells that respond to a particular orientation are arranged in columns perpendicular to the cortical surface and adjacent columns responding to similar orientations^24^, ^25^, ^26^, ^27^, ^28^. The vestibular information involves the static gravitational orientation and cephalic linear accelerations movements, with consequent maintenance of posture and balance^10^^,^^12^^,^^21^. The otolithic organs provide subconscious postural reflexes and contribute to the perception of spatial orientation^29^. Information originated in the otolith organs travel through the vestibulocochlear nerve over the vestibular nuclei to several central nervous system regions to assist the postural control, balance, eye movements coordination and head position^30^^,^^31^. It has been reported that the SVV tilts are a sensitive sign of the vestibular dysfunction, especially the otoliths, and are present in peripheral or central disorders in any location of vestibular pathways, from the labyrinth to vestibular cortex^2^, ^3^, ^4^^,^^21^^,^^32^.

The dynamic SVV test consists of the same exam as the static SVV (adjusting the virtual line in the vertical position without any reference of the real vertical), with addition of continually rotating visual stimulus in the background. It has been described that following a rotation of the peripheral visual field, people experience a sensation of apparent self-motion in general^33^. SVV measurements show that SVV values are tilted during dynamic stimulation in the same direction of the rotation in a stationary observer. Therefore, the dynamic SVV reflects a process of substitution of vestibular signals by visual signals^34^. It has been reported that during space flights the relative contribution of the visual input was enhanced profoundly in microgravity^35^, demonstrating a plasticity in the contribution of the different sensory modalities to the determination of the SVV^5^.

Several clinical studies investigated the influence of different diseases, such as Parkinson's disease, stroke and multiple sclerosis, in the visual perception of verticality^34^^,^^36^, ^37^, ^38^, ^39^, ^40^. SVV tilts after stroke have been shown to be a consequence of lesions involving central vestibular pathways (brainstem, thalamus, cortex), sensory pathways (thalamus, sensory cortex), and lesions in regions concerned with visuospatial analysis such as parietal lesions^36^^,^^37^. Patients with Parkinson's disease present an orientation much more variable than matched-controls and the increased dependence on vision for the SVV task could be related to putamen atrophy present in Parkinson's disease patients^34^^,^^38^. Patients with multiple sclerosis also present abnormal SVV and it could be due to the involvement of brainstem and cerebellar structures that are commonly observed in patients with multiple sclerosis^39^^,^^40^. Patients with sudden unilateral peripheral vestibular dysfunction typically present the SVV deviations to the same side of the vestibular lesion^4^^,^^41^, ^42^, ^43^. It suggests the maintenance of the ocular tilt reaction ipsilateral to the vestibular disorder^4^^,^^41^, ^42^, ^43^. In patients with central dysfunctions, tegmental pontomedullary brainstem lesions cause SVV ipsilateral deviations. In the presence of tegmental pontomesencephalic lesions, contralateral SVV deviations can be observed^44^^,^^45^. Additionally, posterolateral thalamus or parieto-insular vestibular cortex unilateral lesions can cause ipsilateral or contralateral SVV deviations^46^.

The SVV is a widely used modality to assess verticality perception in both research and clinical practice. However several apparatus have been proposed to assess the SVV. Some authors assessed the SVV with a subjective haptic vertical mechanical device composed of a circular background filled with circles and a bar, which the subject have to position in the vertical direction. Named haptic SVV, this method permits the assessment of static SVV (static disk) and dynamic SVV (rotating disk)^1^^,^^5^^,^^47^. However, this method provides somatosensory information that is additional sensory information used to perform the task and, as a consequence, it generates results that do not accurately assess and isolate the involved sensory system. Other device usually used is a laser line projected onto a screen, where the angle of the line's deviation can be read out^36^. Nevertheless, this technique does not allow the dynamic test.

Based on these gaps of previous SVV apparatus, the aim of this study was to develop a virtual system to simplify this evaluation and provide a simple and flexible tool to clinical practice. The software developed in the current study will increase the accessibility to the professionals specialized in otolithic function and human balance disorders, improving the treatment of these affections. In addition, the fact of being a computational tool allows this exam to be explored in different configurations for visual stimulation, and thus new studies could be developed.

## METHOD

### Development of the software

The software development was made in Qt3 which is a C++ class library and a set of tools to build multiplataform GUI (Graphical User Interface) programs, and the interface with the user was elaborated using Qt designer of *Trolltech.* The operating system used was Linux.

The graphical structure of the software was based on the QCanvas class from the Qt library that offers a high level performance for applications that need intensively graphical paintings.

**Figure 1:**
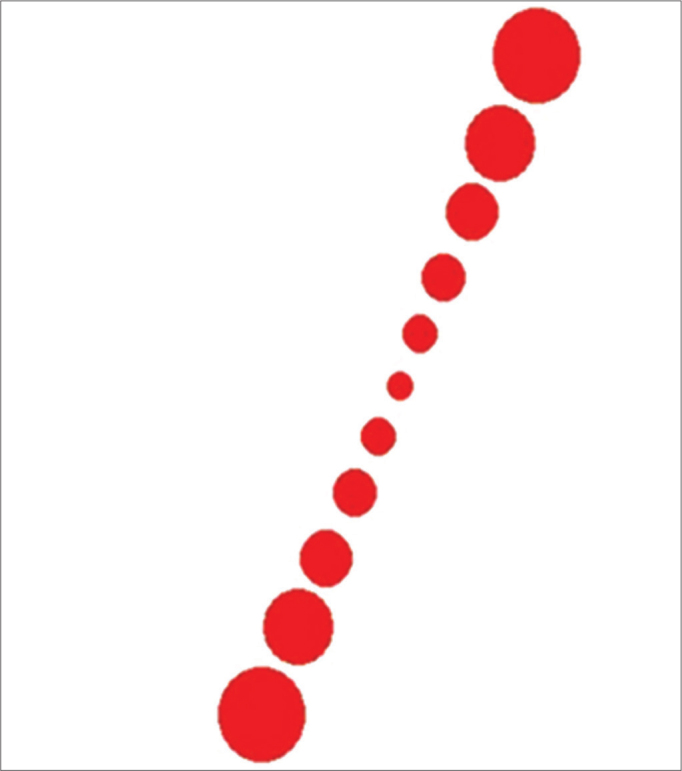
Display of the software interface showing the stimulation environment.

The main stimulation interface (Figure 1) consisted in a white background and a row of seven red circles. The row of seven red circles s always aligned to simulate a line of 11 cm that the subjects are supposed to align in the vertical position. When the line is moved, it rotates in both directions (clockwise and counterclockwise) with the center of rotation localized in the middle of the line.

The use of a row of circles instead of a rectangle or a needle was due to the fact that the tilted line in the monitor is not smooth enough for the application, presenting changing in the geometry according to the tilt angle. These facts could give some clues of the tilt angle and therefore make the whole evaluation biased.

Two extra tabs have been added to the software. The first one was to inform data of the subject that will be examined. The second one was to show the results in degrees of each measurement of an evaluation. The precision for the measurement of the angle was set at 0.1 degree.

### The exam

**Figure 2:**
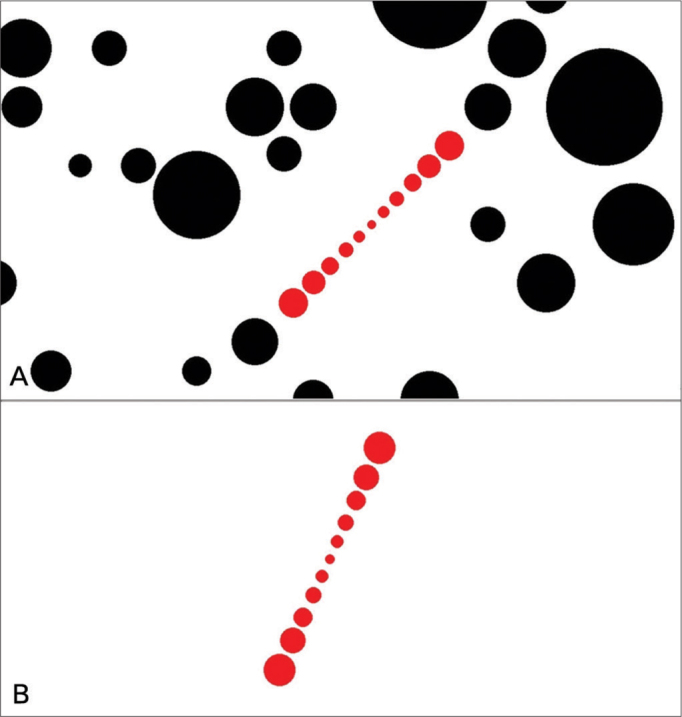
A: The representation of the visual excitation in full screen. For the dynamic condition. B: The representation of the visual excitation in full screen. For the static condition.

**Figure 3:**
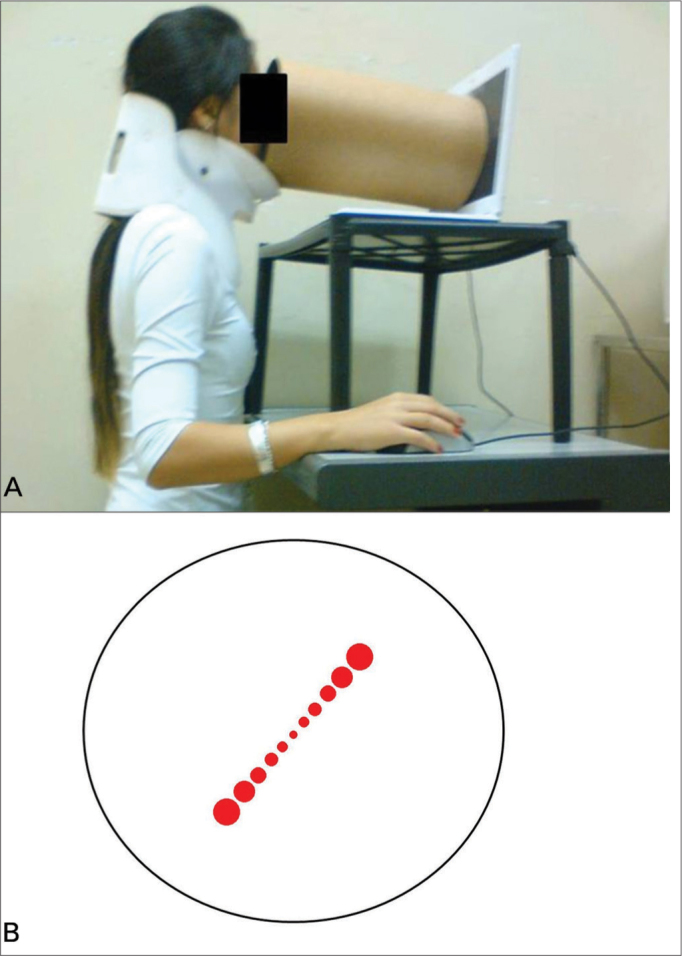
A: The SVV exam is performed in the position shown. B: A dark tube is connected to the monitor so the exam is made with no

The SVV exam consisted in adjusting a virtual line composed by a row of seven red circles in the vertical position using the computer mouse. The right button turned the line into the clockwise (CW) direction, the left one turned into the counterclockwise (CCW) direction and it could be controlled by the either examiner or the subject. The screen was showed in full screen mode (Figure 2 A-B) and a tube was used to deprive the volunteer from any external visual references (Figure 3 A-B). The tube connected the screen to the subject's face and was 30 cm long, with 30 cm of diameter with an opaque black inner part in order to avoid reflectance. This way, the visual angle presented was 20.14° and the exam was also performed in a completely dark room to avoid any visual cue.

The SVV was assessed in both static and dynamic conditions. For the static condition, the screen projected just the row of red circles in a white background with no other visual stimulus. For the dynamic condition, in addition to the row of seven circles in a white background, to provide the dynamic visual stimuli, the screen also projected black circles in random positions and sizes rotating in CW or CCW direction, determined by the examiner. The black circle's angular velocity could be easily changed by the examiner by pressing the keyboard, than its corresponding value was shown in the left upper corner. The up key increased and the down key decreased the angular velocity. In the present study, it was used an angular velocity of 30°/s.

The subjects remained in a seated upright position. They were oriented to rotate the bar using the computer mouse and to inform the examiner when perceived the line in the vertical position. Therefore, when the subject was satisfied with the line's orientation, the examiner executed a command through the keyboard to store the angle deviation and the software automatically random the next initial position of the line. By convention, the angular deviations of the virtual line were defined as positive if tilted CW and negative if tilted CCW in relation to the real vertical. To minimize the learning effect, each subject performed five static SVV measures previous to the real assessment, which were not included in the results of this study. For each condition (static SVV, clockwise dynamic SVV and counterclockwise dynamic SVV), six measures were performed and the final result was determined by the mean value of these measurements^3^^,^^5^^,^^48^^,^^49^. Once they completed the eighteen measurements, a message that the test was finished appeared in the screen and the results were automatically saved in a text form archive with the same name of the evaluated subject.

### Subjects

Thirty healthy volunteers, 23 female (76.7%), aged between 20 and 35 years (mean age 24.17 ± 3.9) performed the SVV exam with a neck brace to prevent cephalic inclinations (Figure 3)^50^. The exclusion criteria were: history of vestibulopathy or any previous sensation of dizziness or vertigo, migraine, neurologic or metabolic disease. Those who wore visual corrective lenses performed the exam using it. All subjects consented to be a part of this project according to the Institution's Ethics Committee under the process number 364/2008.

### Data Analysis

The mean value of the six measurements^3^^,^^5^^,^^48^^,^^49^ was used for the data analyses, which were performed with SPSS (Statistical Package for Social Sciences) Software 17.0 for Windows. After Shapiro-Wilk test, the variables of static SVV and CW dynamic SVV presented normal distribution and were analyzed with Student-t test. The variables of CCW dynamic SVV did not present normal distribution and were analyzed with Mann-Whitney U test. In all tests, the criterion for statistical significance was two-tailed and set at α < 0.05.

## RESULTS

Table 1 presents the mean values and the standard deviation (SD) of both static and dynamic SVV.

**Table 1:** Mean values and SD in each SVV condition

Condition	Deviation (°)
Static SVV	-0.372° ± 1.21
Clockwise Dynamic SVV	1.53° ± 1.80
Counterclockwise Dynamic SVV	-1.11° ± 2.46

SVV: Subjective Visual Vertical.

The mean deviation during the static SVV was −0.372° ± 1.21. During clockwise dynamic SVV, the mean deviation was 1.53° ± 1.80 and during the counterclockwise dynamic condition was −1.11° ± 2.46.

## DISCUSSION

Recently, new methods of vestibular system evaluation were introduced in clinical routine, transforming the investigation of vestibule-ocular reflexes originated on otolithic macula more clarifying^16^. Thus, the acquisition of further information about the otolith end organs functionality generates a more precise diagnosis and consequently proper treatment. Among these assessments, the determination of the SVV is a simple and low cost assessment of otolithic function^16^.

It is well established that normal values of static SVV in the healthy population vary from −2.0° to +2.0°, where the positives signs corresponds to tilts in the clockwise direction and the negative sign, tilts in the counterclockwise direction^4^^,^^10^^,^^11^^,^^51^. Therefore, in the present study, the volunteers presented means that can be considered normal.

For dynamic SVV, it has been already described that, when the subject is in the upright position, the rotatory visual flow with a constant angular velocity causes an angular deviation of the SVV in the same direction as the visual flow^5^^,^^52^. It is believed that after rotation of the peripheral visual field, the individual experiences a sensation of apparent self-motion^33^. Since the SVV deviations of the present study were tilted towards the same direction as the black circles rotation (dynamic stimulus), it is notable that the software developed provokes the same visual flow effect of previous studies^5^^,^^23^^,^^52^ and, therefore, capable to assess dynamic SVV.

However, the analysis of the results obtained in the dynamic SVV is more complex since it involves multivariate cortical processes and the apparatus and protocols used to investigate this perception are not standardized yet. In the literature, the diameter of the disk used to promote the rotational visual stimulus is not established as well as the angular velocity of the background stimulus^5^^,^^23^^,^^43^^,^^47^. It shows that the improvement of standardized protocols in the vertical perception is essential. Otherwise, different results that apparently indicate different processes in the human body are in fact due to differences between the protocol and the equipment used.

The measurement of the SVV is a clinical parameter for the detection of the central and peripheral vestibular diseases and central nervous system lesions^51^^,^^53^. Thus, it is important for clinicians to have appropriate equipment to perform this exam. Recently, a study developed a simple apparatus to perform SVV using a bucket, which was found to be a reliable and simple bedside test^54^. Nevertheless, this SVV evaluation can only be controlled by the examiner, otherwise, haptic information would be provided from the superior limbs if the subject holds the bucket. With the software developed by the current study, it is possible for the subjects to move the virtual line without significant haptic information. Moreover, this software also can easily be used in clinical routines.

An additional advantage is that this SVV software can be attached to a virtual-reality equipment, associating the analysis of static and dynamic paradigms for the motor and sensory systems. Since the perception of verticality interacts with many other systems of the postural control, the possibility of associating the SVV exam to motor and sensory assessments reveals a great interest. Furthermore, this software can also be used for functional Magnetic Resonance Imaging (MRI) studies in SVV. This application involves the employment of MRI to measure the hemodynamic response of the stimulus that, in this case, will be provided by the software. Therefore, this practical software will also enable to identify which brain structures related with the visual vertical perception^55^.

## CONCLUSION

The software developed and described in this study has shown to be practical and accurate to be inserted in the clinical exams routine. Additionally, it has the advantage of potentially be used in conjunction with other diagnostic equipment (e.g. MRI) and it does not provide haptic information to the patient, making the SVV measurement more accurate than several SVV assessment tools available.
